# Effects of Maternal Heartbeat Exposure on Neonatal Autonomic Regulation

**DOI:** 10.3390/pathophysiology33030055

**Published:** 2026-08-03

**Authors:** Sara Puddu, Stefano Vicentin, Filippo Zemin, Paola Veronese, Elisa Cainelli

**Affiliations:** 1 Department of General Psychology, University of Padova, 35131 Padua, Italy; sara.puddu.1@studenti.unipd.it; 2 Institute of Psychology, Clinical Psychology and Psychotherapy in Childhood and Adolescence, University of Osnabrück, 49076 Osnabrück, Germany; stefano.vicentin@uni.osnabrueck.de; 3 Gynaecology and Obstetrics Unit, Department of Women’s and Children’s Health, University Hospital of Padua, 35128 Padua, Italy; filippo.zemin@aopd.veneto.it (F.Z.); paola.veronese@aopd.veneto.it (P.V.)

**Keywords:** autonomous nervous system, heart rate variability, prenatal stress, postnatal, physiological regulation, neonatal, rhythmic stimulation

## Abstract

Background: During intrauterine life, the fetus is continuously exposed to rhythmic sensory stimuli; among these, the maternal heartbeat is one of the earliest and most biologically salient, contributing to the early organization of physiological regulation. This exploratory longitudinal observational study examined whether exposure to the maternal heartbeat influences neonatal autonomic activity and whether this response is moderated by prenatal maternal psychological distress. Methods: Sixteen dyads of mother–child were included in the analyses. Pregnant women were first assessed between 24 and 28 weeks of gestation: maternal distress was measured using the Depression Anxiety Stress Scales—21 (DASS-21), and maternal heartbeat recordings were obtained. At 3–4 weeks postpartum, neonates were exposed to recordings of their own mother’s heartbeat. Neonatal heart rate variability (HRV) was assessed using electrocardiography at rest and during the hearing of the maternal heartbeat. Results: Exposure to the heartbeat significantly reduced high-frequency (HF) HRV compared to baseline (*p* = 0.037), providing evidence for modulation of parasympathetic activity. Importantly, an interaction between condition and maternal distress was observed (*p* = 0.021), suggesting that neonatal autonomic responses differ as a function of maternal psychological state. Conclusions: Overall, these preliminary findings suggest that neonatal autonomic responses to maternal heartbeat exposure may not be uniform but may vary according to maternal psychological distress, highlighting the potential role of the maternal psychophysiological context in shaping early autonomic regulation.

## 1. Introduction

Pregnancy is a period of deep interdependence between mother and fetus [1,2]. Within the intrauterine environment, the fetus is exposed to continuous, repetitive, and multisensory stimulation, including vestibular signals related to maternal movement and posture, tactile input from contact with the uterine walls, variations in light perception despite the low-light environment, and auditory stimuli [3], such as the maternal heartbeat. Fetal auditory capacity develops progressively throughout gestation, with functional maturation beginning in the second trimester [4], and fetal responses to auditory stimuli becoming increasingly organized and differentiated throughout pregnancy. From approximately the third trimester onward, fetuses exhibit measurable changes in heart rate, motor activity, and behavioral states in response to auditory stimulation [5,6], with increasing sensitivity to stimulus frequency and intensity [7,8]. The intrauterine acoustic environment is predominantly characterized by low-frequency, low-amplitude sounds with a repetitive structure, generating a continuous and highly predictable sensory flow [9,10,11]. Familiar stimuli, such as the maternal voice, elicit more stable and specific responses compared to unfamiliar sounds, suggesting the early emergence of sensory discrimination processes [12].

In this context, the temporal dimension of sound represents a central component of prenatal auditory experience, providing a basis for early learning and familiarization with recurring stimuli [9,13,14]. The maternal heartbeat represents one of the earliest and most salient signals in fetal life. Its relatively high intensity (approximately 25 decibels (dB) above background noise) makes it a dominant component of the intrauterine environment [6], providing a continuous rhythmic reference throughout development.

These rhythmic patterns provide a temporal structure that not only supports early learning but also contributes to the modulation of fetal physiological states [11]. The fetus responds consistently to the maternal heartbeat [15], and because this signal varies dynamically according to maternal activity and emotional state, it offers the fetus the opportunity to experience different rhythmic patterns over time [10]. Overall, these findings suggest that the fetus may be considered an active participant in a dynamic process of physiological adaptation, showing sensitivity to variations in maternal rhythms. This capacity may provide a functional basis for the early emergence of attunement and co-regulation processes within the mother–infant dyad, which continue to develop during the postnatal period, as demonstrated by studies highlighting the role of a complex acoustic environment, in which maternal voice, respiration, and bodily signals jointly contribute to the early structuring of the offspring’s regulatory systems [3,13,16].

From this perspective, postnatal regulation does not emerge de novo at birth but develops in continuity with physiological modulation processes already active during prenatal life. Birth does not represent a sudden transition toward full physiological autonomy, but rather an extension of the dependency period that originates in utero [1,17]. The mother’s body and physical presence constitute the primary sources of regulatory input, and through proximity, contact, and interaction, the caregiver provides external regulation that supports the organization of the newborn’s neurobiological, emotional, and behavioral systems [18,19]. Therefore, early interactions are grounded in shared and synchronized physiological processes that support the organization of the infant’s autonomic and emotional systems [20,21].

Practices such as skin-to-skin contact and kangaroo care have been associated with increased stability of heart rate, respiration, and body temperature, as well as improved behavioral organization in the newborn [22,23]. Consistently, exposure to familiar maternal stimuli, such as the maternal voice and heartbeat, has been associated with reduced activation in neonates from the first days of life [24]. These interventions, also widely used in neonatal intensive care settings to support the stabilization of preterm infants [25,26], have been associated with changes in heart rate variability (HRV) [27,28], a sensitive index of autonomic regulation and physiological flexibility [20,29]. In particular, variations in the high-frequency (HF) component of HRV, reflecting parasympathetic activity, have been associated with greater physiological stability and regulatory capacity in the newborn [20,30]. As further evidence of the growing recognition of HRV as an index of autonomic regulation, this measure has increasingly been used to investigate neurodevelopment across infancy, childhood, and adolescence. Individual differences in HRV have been associated with executive functioning, socio-emotional competence, and mental health outcomes, including in at-risk populations, supporting its value as a sensitive and non-invasive measure of developmental functioning [31,32].

This evidence is consistent across developmental stages, with studies reporting associations between HRV and executive functions in children [33], self-regulation during emotional challenges in preschool-aged children [34], and cognitive functioning in adolescents with complex congenital heart disease [35].

However, co-regulation processes are sensitive to several factors, including the maternal psychological state. The perinatal period is characterized by significant neurobiological, endocrine, and emotional changes that, while supporting caregiving behaviors, also increase vulnerability to stress, anxiety, and depressive symptoms [1,36,37]. A large body of research has shown that elevated maternal distress may interfere with the quality of mother–infant attunement and with the regulation of infant physiological states [38,39]. Extending this evidence, maternal mental health during both the prenatal and postnatal periods—including stress, anxiety, and depressive symptoms—is associated with infant autonomic regulation and heart rate variability (HRV) [40,41,42,43].

These findings suggest that maternal signals may not only support but also modulate regulatory processes depending on their quality and stability. Overall, although substantial evidence indicates that maternal signals play a central role in the physiological regulation of the newborn, it remains unclear to what extent specific rhythmic stimuli, such as the maternal heartbeat, directly modulate postnatal autonomic regulation. Furthermore, it is still poorly understood whether this effect varies as a function of maternal psychological state.

The present study aims to examine the effect of exposure to the maternal heartbeat on neonatal autonomic regulation, assessed through HRV indices. Furthermore, we wanted to explore whether this effect is modulated by levels of maternal distress. Based on previous evidence showing that familiar maternal sensory cues generally promote physiological stability and parasympathetic regulation in neonates, we hypothesized that exposure to the maternal heartbeat would be associated with an increase in HF-HRV. Given the limited and inconclusive literature specifically addressing the effects of isolated maternal heartbeat exposure, we also considered this study to have an exploratory component regarding the nature of the autonomic response and its modulation by maternal distress.

## 2. Materials and Methods

### 2.1. Participants

Sixteen mother–infant dyads (14 mothers and 16 newborns, including 2 sets of twins) were enrolled in the study between March 2024 and February 2025 at the outpatient clinic of the Gynecology and Obstetrics Unit, Department of Women’s and Children’s Health, University Hospital of Padua. The small sample size reflects the practical and methodological constraints associated with recruiting pregnant women for studies involving neonatal physiological measurements. Data collection required highly controlled experimental conditions and specialized instrumentation, which limited participant availability. Despite the relatively small sample, the within-subject design enhanced statistical sensitivity by reducing inter-individual variability, allowing each participant to serve as her own control across conditions. Nonetheless, this study remains exploratory, and future studies with larger samples are needed.

Inclusion criteria comprised: absence of current pregnancy-related pathologies or complications; no use of corticosteroid medications; gestational age between the 24th and 28th weeks of gestation; continuous prenatal monitoring with complete documentation of pregnancy, delivery, and neonatal outcomes; completion of demographic and psychological questionnaires; provision of informed consent by both the mother and the infant’s second parent (for neonatal participation); and full compliance with study procedures.

The mothers had a median age of 35 years (31.75; 38) and were assessed during pregnancy at a median gestational age of 24.5 weeks (24; 26). None of the participants reported alcohol consumption or smoking during pregnancy, and none had a psychiatric or neurological diagnosis. Socioeconomic status (SES) was medium in 12/14 cases and low in 2/14 cases.

Infants were born at term; no infant experienced perinatal complications or required resuscitation. Detailed clinical characteristics of delivery and neonatal outcomes are reported in Table 1.

### 2.2. Experimental Procedure

The study employed a two-wave longitudinal design, including a prenatal assessment and a postnatal follow-up.

During the first session (24th–28th week of gestation), participants attended the laboratory, where they were informed about the study procedures. Demographic and anamnestic data were collected, and the Depression Anxiety Stress Scales (DASS-21) questionnaire was administered. Subsequently, maternal heart rate was recorded for 1 min using a Stemoscope device (STEMO200; Shanghai Hulu Devices Co., Ltd., Shanghai, China),while participants were seated at rest in a quiet, sound-attenuated environment. The recording was obtained exclusively to generate the auditory stimulus used during the neonatal session and was subsequently presented as a continuously looped audio track throughout the entire 5-min stimulation period.

The second session took place between the 3rd and 4th week of the infant’s life. Infants were placed on a changing table in a quiet and controlled environment. ECG recordings were acquired after feeding, once infants had naturally fallen asleep. Recordings were initiated only when infants were behaviorally calm, showed no signs of distress, and exhibited minimal body movements to reduce movement-related ECG artifacts. A standard electrocardiography (ECG) recording system was applied to assess heart rate variability (HRV). The experimental protocol included an initial 5-min resting baseline, during which cardiac activity was recorded in the absence of auditory stimulation. Subsequently, an auditory stimulus consisting of the maternal heartbeat recorded during pregnancy was presented. The audio signal was delivered in a continuous loop for 5 min via headphones worn by the infant, with a standardized sound intensity of 65 decibels (dB), a level considered safe for neonatal exposure.

Stimulus presentation and loop control were managed using the OpenSesame software (version 3.3.14). The OpenSesame script was used exclusively for passive auditory stimulus presentation (looped playback of the auditory signal), without additional experimental parameters or task-related manipulations. Auditory stimuli were delivered through EPOS PC 3 CHAT headphones. Cardiac activity was continuously recorded throughout the procedure to assess changes in HRV in response to the maternal auditory stimulus.

Participants also completed questionnaires assessing psychological well-being and underwent a structured interview regarding childbirth and the perinatal period. Data collection was administered via a computerized interface using the secure REDCap platform (electronic data capture platform).

The study was approved by the local Ethics Committee (Prot. n. 24139, 02/2022; 193-c, 01/2024) and conducted in accordance with current ethical guidelines.

### 2.3. Measures

#### 2.3.1. Maternal Psychological Measures

DASS-21: The Depression Anxiety Stress Scales is a self-report questionnaire designed to distinguish core symptoms of depression and anxiety [44]. The short version DASS-21 includes 21 items divided into three subscales: (a) depression, assessing dysphoric mood, low self-esteem, and lack of motivation; (b) anxiety, measuring both somatic and subjective symptoms of anxiety as well as acute fear responses; (c) stress, evaluating irritability, tension, and persistent physiological activation. Each item was rated on a 4-point Likert scale (0–3). Depression, anxiety, and stress subscale scores were computed as the sum of the corresponding items. A global distress index was derived by summing the three subscales.

#### 2.3.2. Neonatal Physiological Measures

HRV: Autonomic nervous system activity was assessed through HRV derived from ECG recordings. Skin preparation was performed at three sites (right and left subclavicular regions and left subcostal region) to ensure optimal electrode adhesion. Ag/AgCl surface electrodes were placed according to Einthoven Lead II configuration.

ECG signals were acquired using a LiveAmp system (Brain Products, Gilching, Germany) under two experimental conditions (resting baseline and auditory stimulation), with a gain of 150, band-pass filtering (0.3–100 Hz), and a sampling rate of 500 Hz (16-bit A/D converter; resolution 0.559 μV/LSB).

ECG recordings were exported to Kubios HRV software (version 2.2) for preprocessing and analysis. R–R intervals were visually inspected to identify artifacts and misdetected beats (e.g., incorrect QRS detections). All identified artifacts were manually corrected on a beat-by-beat basis to ensure accurate reconstruction of the inter-beat interval time series. No automatic artifact correction was applied. In addition, no detrending was performed prior to spectral analysis.

Inter-beat intervals (IBIs) were computed as the time difference (ms) between consecutive R peaks. Frequency-domain HRV analysis was conducted using fast Fourier transform (FFT) applied to the IBI series. Spectral estimates were obtained from artifact-free segments using a 256-s window with 50% overlap. The IBI series was interpolated at 4 Hz prior to spectral analysis.

High-frequency (HF; 0.15–0.40 Hz) power was extracted as the primary index of parasympathetic activity and respiratory sinus arrhythmia (RSA) [30].

HRV indices were computed separately for the resting baseline and auditory stimulation phases using artifact-free segments, allowing comparison of neonatal autonomic regulation across conditions.

### 2.4. Statistical Analyses

A repeated-measures analysis of variance was conducted, with experimental condition (rest vs. heartbeat) as a within-subject factor. A second model, including maternal distress as a covariate, was run. Prenatal distress was further correlated to the HRV in the two conditions to exclude collinearity effects. To quantify condition-related changes in autonomic activity, a difference score (delta HRV) was computed for each participant by subtracting HF-HRV in the rest condition from HF-HRV during the heartbeat condition (delta HRV = heartbeat − rest). Delta HRV was thus correlated with maternal prenatal distress to index the direction of the relationship between maternal distress and infants’ physiological reactivity.

Reliability of the DASS-21 questionnaire was evaluated using Cronbach’s α coefficient.

Statistical analyses were performed using JASP (version 0.19.2).

## 3. Results

### 3.1. Descriptive Results

Overall maternal distress levels, assessed using the DASS-21, were not clinically relevant at the group level (median 4.5 [2; 10.75]; however, by examining individual scores, 5/14 women showed clinically relevant scores. Reliability analysis of the DASS-21 yielded a Cronbach’s α of 0.86.

Descriptive statistics for HF-HRV values across experimental conditions are reported in Table 2. A full set of descriptive statistics for all HRV indices is reported in Supplementary Table S1.

**Figure 1:**
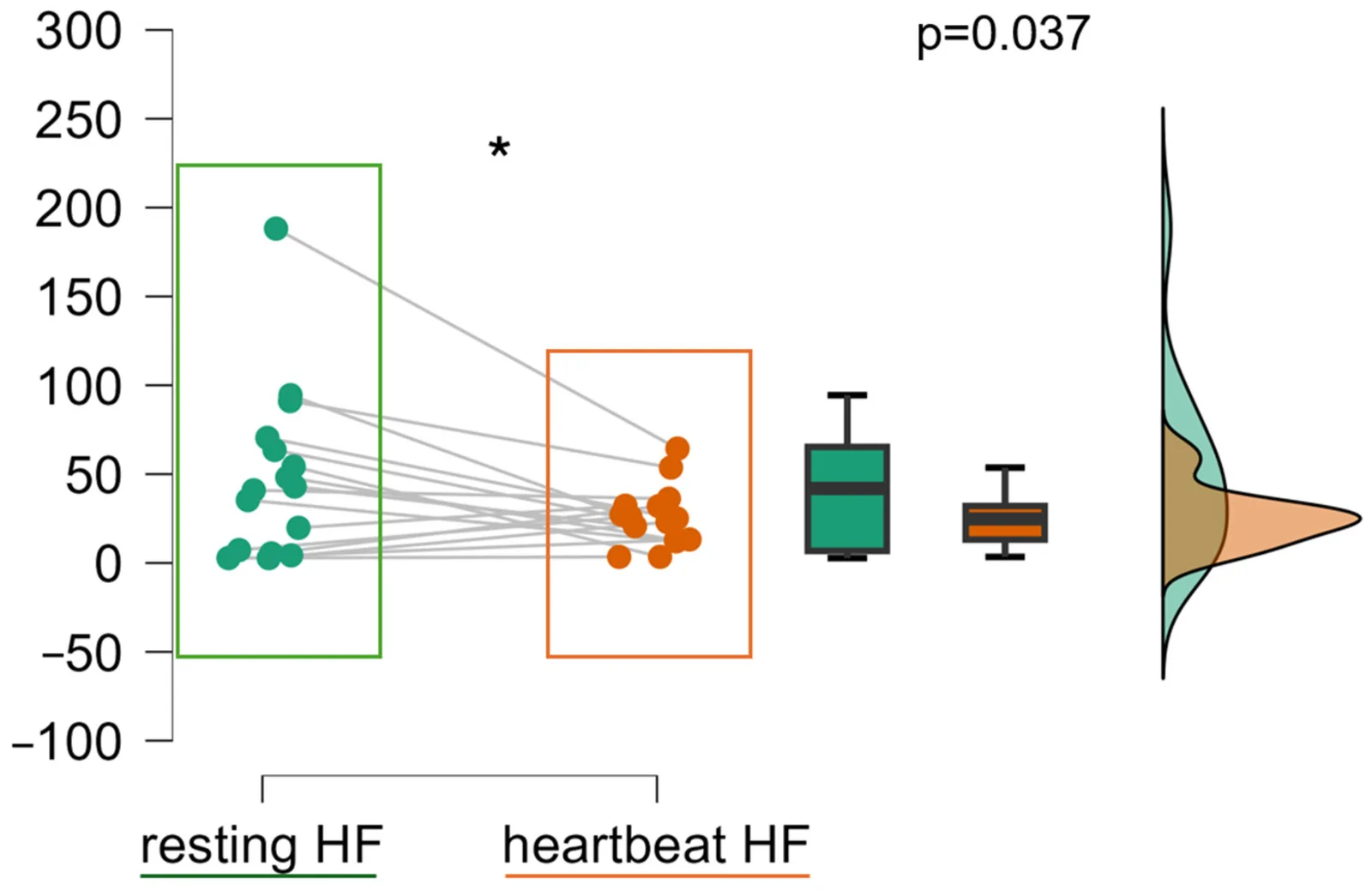
Raincloud plot of HF-HRV values across experimental conditions. Individual data points are shown for the two experimental conditions (resting and heartbeat). Boxplots represent the distribution of the data, and shaded areas represent the data density. * indicates a statistically significant difference between conditions (*p* < 0.05).

The distribution of HF-HRV values across conditions is illustrated in Figure 1, showing individual variability and within-subject differences across experimental phases.

### 3.2. Experimental Effects

The conditions were compared in a within-subject model, where a significant main effect of condition emerged (F(1,15) = 5.265, *p* = 0.037, ω^2^ = 0.074), confirming differences in HF levels across experimental phases, with higher HF values observed in the rest condition (M = 48.23, SE = 12.07) compared to the heartbeat condition (M = 25.64, SE = 4.08). Descriptive statistics indicated that heartbeat HF values were relatively symmetrically distributed (M = 25.64, SD = 16.33; median = 24.81; skewness = 0.93), with no significant deviation from normality (Shapiro–Wilk W = 0.921, *p* = 0.178). In contrast, resting HF values were higher and more variable (M = 48.23, SD = 48.28; median = 41.95), showing a marked positive skew (skewness = 1.68; kurtosis = 3.83) and a significant deviation from normality (Shapiro–Wilk W = 0.835, *p* = 0.008), consistent with the presence of high-value observations (range = 2.74–188.16). However, bootstrap estimates based on 1000 resamples confirmed the stability of these effects. Inspection of standardized residuals indicated no substantial deviation from normality, and no extreme or influential outliers were detected (e.g., standardized residuals within acceptable bounds), supporting the validity of the model despite the non-normal distribution of resting HF.

After the introduction of the covariate, the main effect of the condition was no longer significant (*p* = 0.878). However, a significant interaction between condition and maternal distress was observed (F(1,14) = 6.708, *p* = 0.021, ω^2^ = 0.127), indicating that the effect of condition on HF varies as a function of distress levels. In addition, maternal distress showed a significant between-subjects effect (F(1,14) = 15.982, *p* = 0.001), suggesting an association with overall HF levels. A significant interaction indicated that changes in neonatal HF-HRV across conditions were modulated by maternal distress levels.

To further examine the relationship between maternal distress and infants’ physiological responses, we computed Spearman correlations between maternal distress and HRV separately for each condition. Results showed moderate but non-significant associations both during rest (rho = 0.493, *p* = 0.052) and during heartbeat stimulation (rho = 0.420, *p* = 0.105). Confidence intervals were wide in both cases, indicating substantial uncertainty in the estimates. Overall, these findings suggest that maternal distress was not reliably associated with HRV levels in either condition.

To clarify the direction of the interaction, we examined the association between maternal distress and the change in HF-HRV between rest and stimulation (ΔHRV = heartbeat − rest). Results showed a negative, although non-significant, association (Spearman’s ρ = −0.41, *p* = 0.118), indicating that higher maternal distress was associated with a greater reduction in HF-HRV during stimulation. This pattern is consistent with the interpretation that maternal distress may be linked to increased physiological reactivity in the infant.

## 4. Discussion

The present study examined the effect of exposure to the maternal heartbeat on neonatal autonomic regulation, as well as the role of maternal distress in modulating this response. Initial analyses revealed a reduction in high-frequency (HF) heart rate variability from the resting (baseline) to the heartbeat condition. After including maternal distress as a covariate in the model, a significant interaction between experimental condition and maternal distress emerged, indicating that neonatal reactivity is closely modulated by the mother’s psychophysiological state. Analyzing the direction of the relationship between distress and HRV in the two conditions indicates that maternal distress is associated with a greater reduction in HF-HRV during heartbeat exposure compared to rest.

Contrary to our original hypothesis, which was based on previous evidence suggesting that familiar maternal sensory cues promote parasympathetic regulation, exposure to the maternal heartbeat was associated with a reduction in HF-HRV. Previous studies have reported predominantly calming effects of maternal stimuli—such as reductions in heart rate, energy expenditure, or pain responses [45]. The observed decrease in HF power, traditionally associated with parasympathetic activity and considered an index of respiratory vagal modulation (respiratory sinus arrhythmia, RSA) [30], may be interpreted as a transient reduction in vagal modulation consistent with processes of vagal withdrawal, in line with polyvagal theory [17,20,46]. Within this framework, modulation of the “vagal brake” represents a functional mechanism that enables the transition from a resting state to a state of increased attention and readiness for interaction with the environment. Accordingly, the reduction in HF observed in the present study may reflect a reorganization of the physiological state toward a more functionally activated condition, supporting orientation and adaptation to a biologically and relationally salient stimulus. In fact, although HF-HRV is commonly interpreted as an index of parasympathetic modulation, it reflects a complex interplay of autonomic and respiratory influences.

This interpretation is consistent with developmental evidence indicating that RSA suppression is associated with greater regulatory flexibility and more adaptive responses in social contexts [46,47]. In parallel, research on mother–infant interaction highlights that physiological regulation in early life is inherently dynamic and context-dependent, emerging from processes of mutual coordination and co-regulation rather than reflecting static states of calmness or activation [21,39].

A crucial element for interpreting these findings concerns the nature of the stimulus itself. The maternal heartbeat does not represent a simple acoustic signal, but rather a constitutive feature of the intrauterine environment, to which the fetus is continuously exposed throughout prenatal development. For this reason, it has been described as the “first metronome” of fetal sensory experience [10,48], and can be conceptualized as a stimulus capable of supporting early processes of orientation and relational engagement.

Interestingly, maternal distress emerges as a key factor in shaping neonatal physiological responses. Specifically, after the inclusion of distress in the model, only the interaction between conditions and maternal distress remained significant. This pattern suggests that mother’s psychological state could interact with neonatal responses. This finding aligns with evidence showing a close interconnection between maternal and infant physiological systems, and that maternal stress, anxiety, and depression can influence processes of co-regulation [38,40,41].

One possible neurobiological interpretation is that maternal distress acts as a vulnerability factor, modulating the infant’s threshold of reactivity. This interpretation is broadly consistent with contemporary developmental models suggesting that prenatal maternal stress may influence the development of attentional and regulatory systems, with effects extending into the postnatal period [36,49]. Within this framework, infants of mothers with higher levels of distress may exhibit heightened physiological reactivity to biologically relevant stimuli, as reflected in a greater reduction in HF-HRV. This pattern may indicate altered vagal regulation, potentially reflecting reduced flexibility in modulating autonomic responses to environmental demands.

These findings suggest that neonatal responses to the maternal heartbeat do not follow a uniform or generalizable pattern but rather vary as a function of maternal contextual characteristics. While previous studies have reported predominantly calming effects of maternal stimuli—such as reductions in heart rate, energy expenditure, or pain responses [45]—the present results point to a more nuanced interpretation of neonatal physiological responses. From a developmental perspective, the temporal structure and regularity of physiological signals play a key role in the organization of regulatory processes and dyadic coordination, supporting the infant’s ability to attune to caregiver-derived inputs [21]. In this sense, evidence of real-time physiological synchrony suggests that the coherence of maternal signals may directly shape dyadic regulatory dynamics [50].

Applied to the present context, this perspective suggests that the maternal heartbeat does not constitute a uniformly regulatory input, but rather a signal whose effectiveness depends on its dynamic properties and on the psychophysiological context in which it is embedded. In conditions of elevated maternal distress, the regulatory properties of maternal signals may differ, potentially influencing their salience and the infant’s capacity for physiological attunement. However, the absence of a control auditory condition prevents ruling out the possibility that the observed effects may simply reflect a nonspecific influence of auditory stimulation interacting with maternal distress.

Taken together, these findings support a view of neonatal physiological regulation as a dynamic, context-dependent, and intrinsically relational process. Rather than reflecting a unidimensional state of “calm” or “activation,” variations in autonomic indices appear to express a flexible modulation of the organism’s state as a function of stimulus salience and meaning, emerging from the interaction between signal characteristics, maternal state, and infant responsiveness.

The present findings should be interpreted as preliminary evidence from an exploratory study requiring confirmation in larger and more diverse samples. In particular, the relatively small sample size determined by practical constraints inherent to neonatal research limits the generalizability of the results and the possibility of overfitting and Type I error cannot be ruled out. Therefore, although these findings may be of interest, they should be interpreted with caution. In addition, the study’s ability to detect small effects could be limited. Finally, there are some theoretical issues in this work. First of all, maternal psychological distress was assessed using validated self-report questionnaires rather than standardized clinical diagnostic procedures. Although these instruments are widely used in perinatal research, they may not fully capture clinically significant psychopathology. A further theoretical limitation is the absence of an additional acoustic control condition, which could have strengthened the interpretability of the findings. This was not included due to practical constraints inherent to neonatal research. Specifically, recordings needed to be conducted while infants were naturally asleep and behaviorally calm, but maintaining these conditions for the duration required to administer multiple experimental conditions proved challenging, as many infants awoke before completing the protocol. Consequently, extending the session to include an additional condition was not feasible.

Nevertheless, the present study offers several relevant implications. From a theoretical perspective, it contributes to a more nuanced understanding of early regulatory processes, highlighting how maternal signals operate in a dynamic and context-dependent manner within the dyad. From a clinical perspective, the results suggest that maternal psychological state should not be considered solely as a general risk factor, but also as a variable that may modulate early physiological interactions between mother and infant. If these findings were to be replicated by more rigorous studies, and the role of maternal stress in shaping fetal physiology were confirmed—as previous evidence in the literature appears already to suggest [40,42,49]—they could provide a valuable foundation for the implementation of interventions aimed at supporting maternal well-being, which may have indirect implications for infant physiological regulation.

## 5. Conclusions

In conclusion, the present study provides preliminary evidence suggesting that neonatal autonomic responses to maternal heartbeat exposure may be modulated by maternal psychological distress. These findings should be interpreted with caution given the exploratory design, the limited sample size, and the absence of a control auditory condition. If confirmed by larger and more rigorously controlled studies, they support a view of early physiological regulation as a dynamic and relational process, emerging from the interaction between characteristics of maternal signals, maternal psychophysiological state, and neonatal regulatory capacity within a context-dependent system.

## Figures and Tables

**Table 1:** Clinical characteristics of the offspring and of childbirth.

	Median (Q1; Q3), N/tot
Gestational age, weeks	38 (38; 41)
Days of life at recording	26 (20.75; 32)
Sex (male/female)	10:6
Apgar 1° min	9 (9; 9)
Apgar 5° min	10 (9.75; 10)
Apgar 10° min	10 (9.75; 10)
Reanimation	0/16
Birth weight, g	3267 (2834; 3700)
Birth length, cm	50 (48.5; 50)
Cranial circumference, cm	34.8 (32.75; 35.25)
Apneas	1/16
Infections in the neonatal period	1/16
Type of delivery	
Vaginal	6/14
Cesarean section	3/14
Induction	5/14
Delivery complications	1/14

**Note:** N/tot, number of participants with the characteristic out of the total number of participants included. For maternal variables, the denominator refers to the number of mothers (n = 14), whereas for neonatal variables it refers to the number of infants (n = 16).

**Table 2:** Descriptive statistics of HF-HRV values across experimental conditions.

	Rest HF (ms^2^)	Heart Beat HF (ms^2^)
Median (Q1; Q3)	41.95 (6.79; 65.29)	24.80 (13.15; 32.03)
Mean ± SD	48.22 ± 48.27	25.64 ± 16.32

Note: HF, high-frequency heart rate variability.

## Data Availability

The original data presented in the study are openly available in OSF at https://osf.io/wy87g/overview?view_only=c61669d493084eb9a58c26c121466801 (accessed on 7 July 2026).

## Supplementary Materials

The following supporting information can be downloaded at https://www.mdpi.com/article/10.3390/pathophysiology33030055/s1: Table S1. Descriptive statistics of infant HRV parameters in Rest and Maternal Heartbeat conditions.

## Abbreviations

The following abbreviations are used in this manuscript:

A/D	Analog-to-Digital
ANOVA	Analysis of Variance
dB	Decibels
DASS-21	Depression Anxiety Stress Scales—21
ECG	Electrocardiography
FFT	Fast Fourier Transform
HF	High Frequency
HRV	Heart Rate Variability
IBI	Inter-Beat Interval
Q1	First quartile
Q3	Third quartile
RSA	Respiratory Sinus Arrhythmia
SD	Standard Deviation
SES	Socioeconomic Status
ω^2^	Omega squared
