# Supplementary material for: Effects of Maternal Heartbeat Exposure on Neonatal Autonomic Regulation
Source: Pathophysiology. 2026 Aug 3;33(3):55. doi: 10.3390/pathophysiology33030055 (PMC13510906; doi:10.3390/pathophysiology33030055)
Supplement: Supplementary file 1 [file pathophysiology-33-00055-s001.zip › pathophysiology-4320258-supplementary.pdf]

Supplementary Table S1. Descriptive statistics of infant HRV parameters in Rest and Maternal Heartbeat conditions

	Valid	Missing	Median	Std. Deviation	Minimum	Maximum	25th percentile	50th percentile	75th percentile
Mean RR interval (ms) – Rest	16	0	398.1	40.90	348.7	490.7	359.1	387.9	413.2
SDNN (ms) – Rest	16	0	24.91	13.11	5.950	55.25	17.56	24.86	32.26
VLF power – Rest	16	0	247.4	693.6	14.39	2685	108.3	241.6	562.6
LF power – Rest	16	0	161.5	161.5	11.11	519.9	66.58	131.4	325.7
HF power – Rest	16	0	41.95	48.28	2.740	188.2	5.380	40.89	63.59
LF/HF ratio – Rest	16	0	6.350	7.930	2.420	30.45	4.630	5.940	8.550
Mean RR interval (ms) – Heartbeat	16	0	410.6	101.5	45.81	524.6	376.9	409.3	425.2
SDNN (ms) – Heartbeat	16	0	16.53	10.10	0.880	46.18	12.93	16.06	21.47
RMSSD (ms) – Heartbeat	16	0	7.050	3.746	0.070	15.52	5.680	7.010	8.030
LF power – Heartbeat	16	0	153.3	102.4	26.67	406.8	92.53	145.5	208.3
HF power – Heartbeat	16	0	24.81	16.33	3.430	64.36	12.87	24.54	31.97

Supplementary Table S1. Descriptive statistics of infant HRV parameters in Rest and Maternal Heartbeat conditions

	Valid	Missing	Median	Std. Deviation	Minimum	Maximum	25th percentile	50th percentile	75th percentile
LF/HF ratio – Heartbeat	16	0	4.905	3.354	0.310	12.52	3.240	4.470	7.460

REST = baseline condition; Heartbeat = maternal heartbeat condition. HRV indices are reported separately for each condition.
